# Sequellæ of Rheumatism

**Published:** 1880-03-20

**Authors:** A. G. Hobbs

**Affiliations:** Indiana


### *SEQUELÆ OF RHEUMATISM—A CASE.*

BY A. G. HOBBS, M.D., INDIANA.

J. W——, male, æt. 32, light complexion, short stature and good physique, contracted rubeola fifteen years ago, and during the eruption was exposed to a long cold rain which, he says, "drove in the measles."

In 1868 he had a light attack of rheumatism which lasted him only a few days; another in 1872, more severe, and during the years of 1873, 1874, 1875 and 1876, he had six separate attacks which lasted him from five to twenty days each, each subsequent attack increasing in point of duration and severity.

I first met him in 1877, and having my attention drawn to the pulsation of his carotid arteries, which were visible across the room, I at once, without a critical examination, diagnosed an hypertrophied heart. At this time he was in seeming robust health, but he was not destined to remain so long.

In December, 1877, I was called to see him, when I found him suffering with a severe rheumatism of the knee and ankle joints, the pain soon afterwards reaching the smaller joints of his feet. At the same time he had præcordial pains, lancinating and acute, with dyspnœa and a feeling of impending dissolution.

After quieting him with a hypodermic of ¼gr. morphia, and 1-60 gr. atropia, which was almost magical in its effects, I proceeded to make a physical examination as follows :

*Inspection* revealed a bulging of the præcordial region and the apex beat between the sixth and seventh ribs, two inches to the left of the nipple line.

*Percussion.* A dullness of four inches in diameter extending downward and to left.

*Auscultation.* A murmur heard in diastole, at border of sternum in third intercostal space extending downward and toward apex; the pulse typically "locomotive," visible twenty feet at radius, normal frequency; increased volume; increased power and regular rhythm.

I diagnosed aortic incompetency with compensative hypertrophy. The hypertrophy was enormous.

I now turned my attention to the cardiac pain and think the following facts will sustain me in diagnosing a pericarditis in its inflammatory stage. There was pain, and upon firm pressure over the heart, tenderness; palpitation during paroxysm of pain; increased frequency of pulse; shortness of breath, and pyrexia. At any rate apyretic treatment allayed all pain and tenderness, and thus, I think, aborted the pericarditis in its first stage before effusion took place.

I gave him salicylic acid till he had taken two ounces in eight days. No gastric trouble ensued.

On the ninth day the pains had left his legs and feet, and had attacked his elbows, wrists and fingers, but in a much milder form. I now put him on the alkaline treatment and continued it eight days longer.

On the eighteenth day he had so far recovered as to be able to leave his bed with only a few lingering pains in his fingers. He now rapidly convalesced.

In November, 1878, I was again called to his bedside, when I found him in much the same condition as before, without the pericardial complication. The pains in the joints of his legs and feet were so intense that when the weight of a light cover touched his toes he would shriek with agony.

I at once put him upon alkalies and opium sufficient to slightly allay his pains. For this purpose the latter was required in very large doses. This treatment was continued six days, when he seemed much improved. It now required only small doses of opium to keep him comparatively free from pain. During this time I had lessened the potassa to just a sufficient quantity to keep his urine alkaline.

On the seventh day his pulse, which had always before been full, strong and wiry but normal in frequency, had now become much stronger and was beating 120 times to the minute; temperature normal; the impulse of the heart enormous, and he now for the first time manifested brain symptoms; knew no one except for the instant his attention was riveted; complained of no pain; analysis of the urine revealed both urates and phosphates.

I gave him Norwood's tincture and tincture of aconite 5 m. each every hour, and watched the effect closely. After four doses had been taken his pulse became normal and all brain symptoms passed away, when he complained of slight nausea and a tingling in his fingers, indicating the effects of the aconite. During the next eight days his rheumatism gradually improved, but the above symptoms returned during that period ten times, when each time the veratrum and aconite were administered with the same good results as at first. From this time, he rapidly convalesced and was out on the nineteenth day.

In January, 1879, he had a short attack; the pains intense but vascillating every eight or ten hours from one joint to another; nothing would quiet him but morphia hypodermically, of which I injected ½gr. morning and evening. He was out on the eighth day.

*April*, 1879.—Another seize began which was destined to be the most protracted, if not the most painful of all. This attack began and proceeded as before with essentially the same treatment as before, his heart impulse remaining very strong till about the twelfth day, when it began to intermit and become much weaker, when brain symptoms again ensued. He lay perfectly quiet; eyes most of the time wide open and glaring; pupils dilated; urine passed in large quantities, clear and limpid. Remained in this condition fourteen days, taking digitalis during the whole time with a Dover's powder every six to ten hours. Took his medicine and nourishment mechanically and without resistance.

About the twentieth day his pulse gradually became stronger and more regular, and the mental symptoms slowly passed off. He now complained of pains in his arms and legs as he did upon the day the brain symptoms attacked him, and he spoke of that day as "yesterday." From this date he improved slowly, and in ten days more was attending to slight duties.

*December 1st*, 1879. My patient was again stricken down with his dreaded disease, the pain beginning as before in the joints of his legs and feet, but soon changed to his arms and fingers in a more intense form, if possible, than ever before. This time I kept his urine alkaline with lemon juice, administered while effervescing with bicarbonate of soda. I depended entirely upon the hypodermic syringe to give him rest, as nothing else seemed to produce the least effect, not even opium in large doses by the stomach. As a rule it required ½gr. morphia night and morning, but frequently I was compelled to increase the dose to ¾ and sometimes to 1 gr. at night, before the desired rest could be obtained. He remained in this condition about three weeks with the excepton of remissions about every five or six days, the severity gradually lessening for about that period, when, all at once, the pains returned as severe as before.

During the fourth week, while upon the chamber, he had an attack of syncope and fell prostrate to the floor; from this condition he passed into a coma, from which he could be aroused when loudly spoken to, but immediately closed his eyes again. I found him in this condition two hours afterwards; his heart was fluttering and intermittent; radial pulse almost imperceptible. I injected 1-20 gr. atropia, and in thirty minutes he was laughing and talking of his fall.

He is now (January 20th) attending to his drug store. I neglected to state that in all of his attacks his joints were wrapped in cotton wool and sometimes saturated in a liniment.

In this report I have curtailed my notes as much as I thought compatible with a full understanding of the case, and without making a lengthy recapitulation, I will call the attention of the reader to only a few points.

During the interim of his attacks he enjoys good health, his hypertrophied heart causing him no trouble whatever. On the contrary, it seems to be perfectly compensative, and since hypertrophy to be compensative must be in direct ratio with the increased resistance to the blood current, the resistance or obstruction in this case certainly is very great. At one time he presents all the symptoms of cerebral congestion from the blood being driven with such great force into the cerebral arteries, and notice here how rapidly and effectively arterial sedatives reduced the brain symptoms.

Again he presents all the symptoms of cerebral anemia with a weak and almost imperceptible pulse, which is so unusual with his hypertrophied heart. Digitalis, as a heart tonic, did not display the happy effect that was secured by the sedatives. There could have been no breaking down of the hypertrophy to cause this long weakening of the heart, because after remaining in this condition about fourteen days the impulse became as strong as before and has remained so.

His attack of syncope from a sudden exertion, with the fluttering and intermitting heart looked like heart failure, but why should there have been heart failure without degeneration of its walls? There has been no indications of degeneration since.

The almost magical effect of atropia is confirmatory of our first impression—that there was a threatening of heart failure.

It will be seen that salicylic acid did not prove a specific in this case, nor could it be said with certainty that the alkaline treatment exerted any great influence, for, according to the report of Bellevue and Kings College Hospitals, the mint water—in other words the placebo treatment—effects about the same results in these cases as do other modes. What then are we to do in these cases? Let the rheumatism alone, and treat the complications? Of course, we owe it to our patient to give him as much comfort as possible, and in my judgment nothing will do it like the hypodermic syringe, and we must not stop with the textbook doses, but increase the morphia till we find the amount that is necessary to give ease, even to the extent of 1 gr. if a less quantity does not accomplish this end.

